# Body composition and gene expression QTL mapping in mice reveals imprinting and interaction effects

**DOI:** 10.1186/1471-2156-14-103

**Published:** 2013-10-29

**Authors:** Ye Cheng, Satyanarayana Rachagani, Angela Cánovas, Mary Sue Mayes, Richard G Tait, Jack CM Dekkers, James M Reecy

**Affiliations:** 1 Department of Animal Science, Iowa State University, 2255 Kildee, Ames, IA, USA; 2 Current address: Department of Biochemistry and Molecular Biology, Department of Pathology and Microbiology, Eppley Institute for Research in Cancer and Allied Diseases, University of Nebraska Medical Center, Omaha, NE, USA; 3 Genètica i Millora Animal, Institute de Recercai Tecnologia Agroalimentària, Lleida, Spain

**Keywords:** eQTL mapping, QTL mapping, Body composition, Myostatin, Imprinting, Interaction, Mouse

## Abstract

**Background:**

Shifts in body composition, such as accumulation of body fat, can be a symptom of many chronic human diseases; hence, efforts have been made to investigate the genetic mechanisms that underlie body composition. For example, a few quantitative trait loci (QTL) have been discovered using genome-wide association studies, which will eventually lead to the discovery of causal mutations that are associated with tissue traits. Although some body composition QTL have been identified in mice, limited research has been focused on the imprinting and interaction effects that are involved in these traits. Previously, we found that *Myostatin* genotype, reciprocal cross, and sex interacted with numerous chromosomal regions to affect growth traits.

**Results:**

Here, we report on the identification of muscle, adipose, and morphometric phenotypic QTL (pQTL), translation and transcription QTL (tQTL) and expression QTL (eQTL) by applying a QTL model with additive, dominance, imprinting, and interaction effects. Using an F2 population of 1000 mice derived from the *Myostatin*-null C57BL/6 and M16i mouse lines, six imprinted pQTL were discovered on chromosomes 6, 9, 10, 11, and 18. We also identified two IGF1 and two Atp2a2 eQTL, which could be important trans-regulatory elements. pQTL, tQTL and eQTL that interacted with *Myostatin*, reciprocal cross, and sex were detected as well. Combining with the additive and dominance effect, these variants accounted for a large amount of phenotypic variation in this study.

**Conclusions:**

Our study indicates that both imprinting and interaction effects are important components of the genetic model of body composition traits. Furthermore, the integration of eQTL and traditional QTL mapping may help to explain more phenotypic variation than either alone, thereby uncovering more molecular details of how tissue traits are regulated.

## Background

With respect to complex traits (i.e., phenotypes controlled by multiple genes), although people are still doubting the importance of epistasis or gene by gene interation [1], there is strong evidence that epistasis should not be neglected when studying complex traits [2-4]. For example, in mammals, coat color is controlled by interactions among several genes [5]. Furthermore, Brockmann et al. (2000) reported that epistasis could account for approximately 33-36% of the phenotypic variance observed in body weight and fat accumulation and 20-33% of the variance in muscle weight and hormone serum concentrations in mice. These results highlight the important role of epistasis in the control of phenotypic traits. In addition to the epistasis, imprinting effect is another important contribution to the phenotypic variance in complex traits. Cheverud et al. suggested that combing phenotype-based mapping and bioinformatics approaches could help to understand the mechanisms that underlie imprinting [6].

It has been recognized that epistasis exists between modifier genes and major genes, such as *Myostatin*, to impact the expressivity of muscling phenotype. *Myostatin* variants have been shown to enhance muscle growth in cattle, dogs, mice, and humans [7-10]. In contrast to breeds like Belgian Blue, some homozygous *Myostatin*-null South Devon cattle do not exhibit the double-muscling phenotype [11]. Furthermore, we and others have reported the identification of QTL that interact with *Myostatin* to control growth and muscling in mice [12-14].

Multiple genomic regions associated with growth and fatness were identified in pigs [15]. In humans, genetic variation present in several chromosomal regions has been associated with obesity traits [16-18]. Unfortunately, the functional genes involved in body composition in these regions have not yet been identified. It has been pointed out that transcriptome mapping [19] might be a new method to identify other loci that control body composition [20]. Transcriptome mapping, also called “genetical genomics”, was first proposed by Jansen and Nap [21]. They suggested that traditional quantitative genetic approaches could be applied to genome-wide gene expression data as a valuable approach towards the identification of regulatory regions. This concept has been successfully applied in more than a dozen species, including mouse, maize, human, rat, eucalyptus, and *Arabidopsis thaliana*[22-33]. Pomp et al. suggested that transcriptome mapping might provide details about the molecular mechanism of obesity QTL [20]; for example, a QTL may be identified as a *trans*- or *cis*-regulator based on its physical distance from the targeted gene.

In this study, we performed an extensive QTL mapping experiment designed to evaluate multiple layers of genetic regulation of body composition traits through the identification of phenotypic QTL(pQTL), translation and transcription QTL (tQTL), and expression QTL (eQTL). We used an F2 population from the M16i mouse line and C57BL/6 *Myostatin*-null mouse line. M16i is a polygenic obese mouse line that was derived from an ICR mouse line after selection for 3–6 week high body weight gain [34]. M16i mice exhibit many typical obesity phenotypes [35-38]. In contrast, the *Myostatin*-null mouse displays a significant decrease in body fat accumulation with a massive increase in skeletal muscle mass [39].

We measured ten muscle, adipose, and morphometric phenotypes, six transcription and translation traits, and nine gene expression traits. The nine genes studied here were chosen based on the differentially expressed genes in skeletal muscle from *Myostatin*-null versus *Myostatin* wild-type mice that were identified from our previous microarray experiment [40]. Additive, dominance, and imprinted QTL models were evaluated with the aim of identifying potential QTL. Interaction effects between QTL and the *Myostatin* genotype, reciprocal cross, and sex were evaluated as well. In addition, the amount of phenotypic variation accounted for by each QTL was computed. Combined with the other growth trait QTL that were identified in our previous study, these results provide further information about how genetic variants regulate body composition.

## Results

### Data evaluation

Summary statistics for all phenotypic measurements are presented in Additional file 1: Table S1, and pairwise phenotypic correlations are presented in Additional file 1: Table S2 and Additional file 2: Table S7. We observed high correlations between most traits. For example, the two adipose traits, adiposity index (AI) and fat pad weight percentage (FAT), were significantly correlated (P < 0.05) with all other traits.

The significant main and interaction effects identified with PROC GLM were included as fixed effects in the QTL model (Additional file 1: Table S3). In addition, imprinting effects were also included. Details of these models are discussed in the Methods section.

### Additive and dominance effects

We identified 20 and 40 non-imprinted QTL at 1% and 5% genome-wide significance levels, respectively, using an additive and dominance QTL model (Table 1). Among these 40 QTL, 38 were pQTL and two were eQTL.

**Table 1:** Statistics of non-imprinted QTL

			**Peak**	**Flanking markers**^**c**^				**Estimate**^**d**^				
**Chr**	**Trait**^**a**^	**Groups**	**(cM)**^**b**^	**Left**	**Right**	**F-value**	**LOD**	**a**	**s.e**_**a**_	**d**	**s.e**_**d**_	**% var**^**e**^
1	Gastro**	pQTL	23	rs3696088	rs13472794	35.40	14.84	-0.0417	0.0054	-0.0239	0.0073	6.73
1	Pec**	pQTL	23	rs3696088	rs13472794	53.84	22.19	-0.0084	0.0089	-0.0503	0.0120	9.88
1	Tnni1	eQTL	23	rs3696088	rs13472794	8.73	3.75	-0.3433	0.0965	-0.2555	0.1282	2.23
1	AI**	pQTL	24	rs3696088	rs13472794	13.00	5.57	0.0813	0.0165	0.0332	0.0219	2.64
1	Fat**	pQTL	24	rs3696088	rs13472794	13.54	5.80	0.0817	0.0167	0.0367	0.0222	2.68
1	Edl**	pQTL	25	rs3696088	rs13472794	7.19	3.10	-0.0327	0.0086	0.0010	0.0112	1.44
2	Edl	pQTL	78	rs3144393	rs13476878	6.79	2.93	-0.0179	0.0052	0.0107	0.0082	1.36
2	Gastro**	pQTL	80	rs3144393	rs13476878	9.40	4.04	-0.0117	0.0033	0.0124	0.0053	1.88
2	AI**	pQTL	88	rs3144393	rs13476878	18.65	7.95	0.0519	0.0091	-0.0263	0.0138	3.65
2	Fat**	pQTL	88	rs3144393	rs13476878	19.62	8.35	0.0535	0.0092	-0.0279	0.0140	3.84
3	Pec	pQTL	44	rs13477174	rs3670634	9.84	4.23	0.0143	0.0049	0.0243	0.0069	1.96
3	Gastro	pQTL	56	rs3663873	rs13477430	6.51	2.81	0.0084	0.0032	0.0134	0.0050	1.31
3	Edl	pQTL	64	rs3663873	rs13477430	5.45	2.35	0.0179	0.0058	0.0145	0.0103	1.10
4	IGF1	eQTL	68	rs6324470	rs3659226	8.46	3.63	0.0985	0.0312	-0.1273	0.0516	2.17
5	Gastro	pQTL	49	rs6256504	CEL-5_52953963	4.51	1.95	-0.0050	0.0030	0.0113	0.0048	0.91
6	Gastro	pQTL	0	-	rs13478602	6.64	2.86	-0.0058	0.0028	0.0125	0.0041	1.33
6	AI**	pQTL	27	rs13478727	rs13478839	10.27	4.41	0.0393	0.0090	-0.0121	0.0136	1.99
6	Fat**	pQTL	28	rs13478727	rs13478839	9.97	4.29	0.0394	0.0091	-0.0122	0.0137	1.98
6	lengthNT**	pQTL	45	rs3676254	rs3656205	13.45	5.76	0.2921	0.0578	0.1200	0.0975	2.58
7	Gastro	pQTL	47	rs3676254	rs3656205	5.65	2.44	0.0227	0.0068	-0.017	0.0073	1.14
7	Pec**	pQTL	47	rs3676254	rs3656205	10.54	4.53	0.0445	0.0113	-0.0169	0.0122	2.10
8	Gastro	pQTL	37	rs13479657	rs13479757	7.22	3.11	0.0134	0.0036	-0.0032	0.0060	1.45
8	Fat**	pQTL	68	rs3678433	rs6182338	15.00	6.42	0.0556	0.0102	0.0022	0.0163	2.96
8	AI**	pQTL	69	rs3678433	rs6182338	15.38	6.58	0.0546	0.0100	0.0032	0.016	2.95
9	Gastro	pQTL	0	-	rs13480071	4.28	1.85	-0.0039	0.0029	-0.0100	0.0039	0.86
9	AI	pQTL	23	rs8259427	rs6213724	8.94	3.85	0.0311	0.0091	0.0334	0.0139	1.79
9	Fat	pQTL	23	rs8259427	rs6213724	9.18	3.95	0.0320	0.0091	0.0336	0.0140	1.83
10	lengthNT	pQTL	26	rs13480578	CEL-10_58149652	7.96	3.43	0.1970	0.0519	-0.0946	0.0792	1.58
10	Gastro	pQTL	30	rs13480579	CEL-10_58149653	4.50	1.94	-0.0082	0.0028	-0.0033	0.0041	0.91
11	Tail**	pQTL	25	rs6276300	rs6199956	16.87	7.21	0.2461	0.0425	0.024	0.0682	3.29
11	lengthNT**	pQTL	26	rs6276300	rs6199956	21.55	9.16	0.3596	0.0556	0.0833	0.0892	4.15
11	BMI	pQTL	49	rs13481054	rs3701609	8.23	3.55	0.5312	0.1385	0.2684	0.1978	1.64
11	Gastro	pQTL	68	rs3653651	rs13481216	4.86	2.10	0.0086	0.0028	0.0026	0.0040	0.98
14	Gastro	pQTL	34	rs8251329	rs3712401	7.77	3.35	0.0118	0.0030	0.0030	0.0042	1.56
17	AI**	pQTL	17	rs13482893	rs3719497	11.2	4.81	0.0418	0.0091	-0.0079	0.0145	2.14
17	Fat**	pQTL	17	rs13482893	rs3719497	10.94	4.70	0.0415	0.0091	-0.0097	0.0144	2.14
17	Edl**	pQTL	33	rs3023442	rs6395919	7.74	3.34	-0.0187	0.0049	0.0072	0.0079	1.55
17	Gastro**	pQTL	68	rs6257479	rs3663966	9.62	4.14	-0.0128	0.0029	0.0024	0.0042	1.92
18	lengthNT	pQTL	34	rs3670254	rs3718618	8.39	3.62	0.2128	0.0522	0.0373	0.078	1.67
18	lengthNA	pQTL	35	rs3670254	rs3718618	8.62	3.72	0.1345	0.0325	0.0149	0.0486	1.71

^a^*Trait abbreviations*: *lengthNA* nasal to anal length (cm), *lengthNT* nasal to tail length (cm), *AI* adiposity index, *BMI* body mass index, *Tail* tail length (cm), *Soleus* soleus muscle weight percentage, *Gastro* gastrocnemius muscle weight percentage, *Edl* EDL muscle weight percentage, *Pec* pectoralis muscle weight percentage, *Fat* average gonadal fat pad weight percentage (epididymal for males and perimetrial for females). *Tnni1* troponin I type 1 expression, *IGF1* insulin-like growth factor 1 expression. QTL with an F-value that exceeded 1% genome-wide permutation threshold are denoted by **; QTL without ** exceeded 5% genome-wide permutation threshold.

^b^Peak position of QTL detected in Kosambicentimorgans.

^c^Flanking markers (left and right) of the QTL peak. A“-“ notation denotes the end of the chromosome. See Additional file 1: Table S6 for marker information.

^d^a: additive effect; s.e_a_: standard error of additive effect; d: dominance effect; s.e_d_: standard error of dominance effect.

^e^% var: percentage of phenotypic variance that a given QTL position could account for.

We detected pQTL for all ten phenotypic traits measured in this study, except for the soleus muscle weight. The greatest number of pQTL was associated with gastrocnemius weight, while only one pQTL each was detected for body mass index (BMI) and tail length, both located on chromosome 11. The 38 non-imprinted pQTL were distributed across 13 chromosomes. No pQTL were identified on chromosomes 4, 12, 13, 19, and X. Chromosome 1 harbored the greatest number of pQTL. The phenotypic variation accounted for by these 38 pQTL ranged from 0.86% to 9.88%. Interestingly, the pQTL that were associated with pectoralis and gastrocnemius weights on chromosome 1 had the two largest F-values. These two pQTL also explained the largest amount of phenotypic variation (Table 1). QTL tended to have larger additive than dominance effects, although most additive and dominance effects were not large.

Two eQTL were identified at a 5% genome-wide significance level (Table 1). The eQTL on chromosome 1 was associated with Tnni1 expression level, whereas the eQTL on chromosome 4 impacted IGF1 expression. Both eQTL explained about 2% of the phenotypic variation. In this context, we have found positional concordance between eQTL located on chromosome 1 (23 cM) associated with Tnni1 expression level and two pQTL associated with pectoralis and gastrocnemius weight, which were located in the same chromosomal region.

No significant additive or dominance effects were identified for the six transcription and translation traits at a 5% genome-wide significance level.

### Imprinting effect

**Figure 1:**
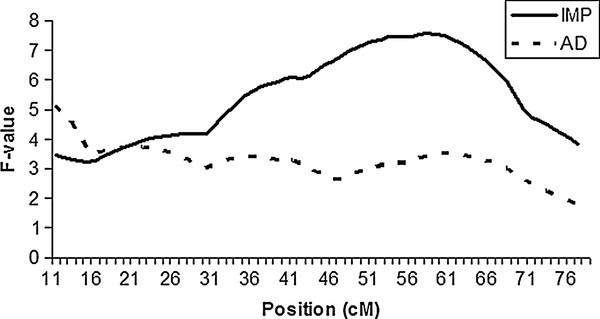
**Identification of an imprinted pQTL on chromosome 10 at 58 cM that controls nasal to anal length.** IMP: imprinted QTL model. AD: additive and dominance QTL model. Vertical line indicates the position of the imprinted QTL.

Imprinted QTL with a comparison-wise *P*-value of less than 0.05 were only detected for phenotypic traits (Table 2). These pQTL were located on chromosomes 6, 9, 10, 11, and 18. Among these imprinted pQTL, three were associated with nasal to anal length. The two imprinted pQTL on chromosome 18 shared the same peak position and were both associated with adipose traits. The amount of variation accounted for by these pQTL was very similar and ranged from 2.2-2.4% of the total phenotypic variation. Theimprinted pQTL on chromosome 10 for nasal to anal length was plotted in Figure 1. In general, the *P*-values associated with additive pQTL were more significant than those associated with dominance and imprinted pQTL (Additional file 1: Table S4).

**Table 2:** **Statisticsof imprinted QTL with comparison-wise *P*-value < 0.05**

			**Peak**^**b**^	**Flanking markers**^**c**^		**Estimate**^**d**^						
**Chr**	**Trait**^**a**^	**Groups**	**(cM)**	**Left**	**Right**	**a**	**s.e**_**a**_	**d**	**s.e**_**d**_	**i**	**s.e**_**i**_	**% var**^**e**^
6	lengthNA	pQTL	45	rs4226048	mCV24115224	0.1438	0.0361	0.0867	0.0609	-0.1492	0.0631	2.21
9	Edl	pQTL	1	rs13480071	rs13480109	-0.0008	0.0046	-0.0080	0.0064	0.0528	0.0114	2.27
10	lengthNA	pQTL	58	rs13480754	rs13480776	0.0679	0.0320	0.0693	0.0478	0.2329	0.0588	2.24
11	lengthNA	pQTL	23	rs6276300	rs6199956	0.1488	0.0350	0.0921	0.0561	-0.1188	0.0566	2.48
18	AI	pQTL	39	rs3670254	rs3718618	0.0406	0.0093	-0.0114	0.0141	0.0315	0.0156	2.42
18	Fat	pQTL	39	rs3670254	rs3718618	0.0411	0.0094	-0.0114	0.0142	0.0323	0.0157	2.36

^a^Trait abbreviations are the same as in Table 1.

^b^Peak position of QTL detected in Kosambi centimorgans.

^c^Flanking markers (left and right) of the QTL peak. See Additional file 1: Table S6 for marker information.

^d^a: additive effect; s.e_a_: standard error of additive effect; d: dominance effect; s.e_d_: standard error of dominance effect; i: imprinting effect; s.e_i_: standard error of imprinting effect.

^e^%var: percentage of phenotypic variance that a given QTL position can account for.

### Interactions with *Myostatin* genotype, reciprocal cross, and sex

We identified 19 chromosomal positions that significantly interacted with *Myostatin* genotype (comparison-wise *P*-value < 0.05) (Table 3). In addition, another 20 and 16 QTL positions were detected that significantly interacted with sex and reciprocal cross, respectively (Tables 4 and 5). The first model (am + dm + im) tested for additive, dominance, and imprinted QTL by *Myostatin* genotype effects. The second model (am + dm) tested additive and dominance QTL by *Myostatin* genotype effects. The third model (am) estimated the *P*-value of the additive QTL by *Myostatin* genotype effect. A majority of the QTL that interacted with *Myostatin* genotype or sex were associated with adipose traits (Tables 3 and 4). Most of the QTL by *Myostatin* genotype, sex, or reciprocal cross interactions were additive or dominant. One exception was that of the BMI pQTL on chromosome 14 that significantly interacted with sex, which appeared to behave in an imprinted fashion. Interestingly, this same chromosomal region interacted with *Myostatin* genotype, but in a dominant manner. Another exception was a tail length pQTL on chromosome 7 that interacted with reciprocal cross in an imprinted fashion.

**Table 3:** **Statistics of QTL that interact with *Myostatin *** **genotype**

			**Position**	**Flanking markers**^**c**^		**am + dm + im**^**d**^		**am + dm**^**e**^		**am**^**g**^	
**Chr**	**Trait**^**a**^	**Groups**	**(cM)**^**b**^	**Left**	**Right**	***P*-value**	**%var**	***P*-value**	**%var**	***P*-value**	**% var**
1	BMI	pQTL	22	rs3696088	rs13472794	7.35E-02	0.70	*3.85E-02*	0.65	*8.48E-03*	0.69
1	Gastro	pQTL	24	rs3696088	rs13472794	*2.45E-08*	3.58	*1.77E-08*	3.34	1.49E-01	0.20
1	Pec	pQTL	25	rs3696088	rs13472794	*3.95E-12*	5.06	*1.08E-11*	4.56	*3.52E-03*	0.80
2	P/D	tQTL	61	rs13476636	rs3144393	*2.66E-04*	2.64	*1.56E-04*	2.43	*1.52E-04*	2.00
3	Pec	pQTL	43	rs13477174	rs3670634	*2.31E-02*	0.95	*1.02E-02*	0.91	8.95E-02	0.29
3	Atp2a2	eQTL	120	rs3724562	CEL-3_159340478	*8.87E-03*	1.57	*7.31E-03*	1.34	*3.72E-03*	1.14
6	Fat	pQTL	27	rs13478727	rs13478839	*4.24E-03*	1.31	*1.36E-03*	1.31	*2.74E-04*	1.31
6	lengthNT	pQTL	69	UT_6_123.37228	rs3688358	1.34E-01	0.55	7.28E-02	0.52	*3.65E-02*	0.43
8	AI	pQTL	20	rs13479657	rs13479757	*1.42E-02*	1.04	*5.09E-03*	1.04	*1.14E-03*	1.04
8	Fat	pQTL	20	rs13479657	rs13479757	*1.15E-02*	1.10	*4.13E-03*	1.09	*9.01E-04*	1.09
8	Igf2	eQTL	33	rs13479657	rs13479757	6.63E-02	1.10	*3.30E-02*	1.05	8.20E-01	0.01
14	BMI	pQTL	63	rs3709178	rs13482404	9.57E-02	0.63	*4.50E-02*	0.62	1.23E-01	0.24
17	Fat	pQTL	15	rs13482893	rs3719497	*4.84E-02*	0.74	*3.38E-02*	0.65	*1.44E-02*	0.64
17	AI	pQTL	28	rs3023442	rs6395919	*3.76E-02*	0.83	*2.68E-02*	0.71	*7.06E-03*	0.71
17	Soleus	pQTL	69	rs6257479	rs3663966	*3.77E-02*	0.86	*1.82E-02*	0.81	*3.49E-02*	0.45
18	AI	pQTL	42	rs3718618	rs13483438	*3.92E-02*	0.82	*2.97E-02*	0.69	*4.65E-02*	0.39
18	Fat	pQTL	42	rs3718618	rs13483438	*3.27E-02*	0.87	*2.78E-02*	0.71	*4.88E-02*	0.39
X	Atp2a2	eQTL	54	rs13484003	rs13484087	*1.74E-04*	2.69	*1.11E-04*	2.46	*1.96E-03*	1.30
X	Egf	eQTL	56	rs13484003	rs13484087	*3.34E-02*	1.18	*1.75E-02*	1.10	*1.33E-02*	0.83

^a^Trait abbreviations are the same as in Table 1. P/D: total protein/total DNA. Atp2a2: ATPase, Ca++ transporting, cardiac muscle, slow twitch 2 expression. Igf2: insulin-like growth factor 2 expression. Egf: epidermal growth factor expression.

^b^Peak position of QTL detected in Kosambi centimorgans.

^c^Flanking markers (left and right) of the QTL peak. See Additional file 1: Table S6 for marker information.

^d^am + dm + im tested the overall interaction, which included additive, dominance, and imprinted pQTL by *Myostatin* genotype interactions. *P*-value < 0.05 is shown in italics %var: percentage of phenotypic variance accounted for at QTL position.

^e^am + dm tested for non-imprinted interactions, which included additive and dominance pQTL by *Myostatin* genotype interactions. *P*-value < 0.05 is shown in italics %var: percentage of phenotypic variance accounted for at QTL position.

^f^am tested for additive interactions, which included additive pQTL by *Myostatin* genotype interactions. *P*-value < 0.05 is shown in italics % var: percentage of phenotypic variance accounted for at QTL position.

**Table 4:** Statistics of QTL that interact with sex

			**Position**	**Flanking markers**^**c**^		**am + dm + im**^**d**^		**am + dm**^**e**^		**am**^**f**^	
**Chr**	**Trait**^**a**^	**Groups**	**(cM)**^**b**^	**Left**	**Right**	***P*-value**	**%var**	***P*-value**	**% var**	***P*-value**	**% var**
1	Soleus	pQTL	19	rs3696088	rs13472794	*1.57E-06*	2.94	3.28E-01	0.23	5.05E-01	0.05
1	Pec	pQTL	23	rs3696088	rs13472794	*1.23E-03*	1.45	*5.64E-03*	0.95	*7.01E-04*	1.07
1	Edl	pQTL	89	rs3666905	rs13476312	*1.65E-02*	1.03	3.48E-01	0.21	1.68E-01	0.19
2	P	tQTL	34	rs6268714	rs13476554	*1.18E-02*	1.53	*3.73E-03*	1.55	2.04E-01	0.23
3	Pec	pQTL	36	rs13477132	rs13477174	*1.90E-02*	0.99	8.37E-02	0.50	*2.39E-02*	0.51
3	Edl	pQTL	64	rs3663873	rs13477430	*4.79E-02*	0.80	*2.09E-02*	0.78	4.70E-01	0.05
6	EGF	eQTL	32	rs13478839	rs4226048	*4.18E-03*	1.79	*2.08E-03*	1.67	*4.85E-04*	1.64
6	Fat	pQTL	10	petM-02094-1	rs3678887	*3.61E-02*	0.86	*2.06E-02*	0.78	3.46E-01	0.09
6	AI	pQTL	11	petM-02094-1	rs3678887	6.05E-02	0.73	*3.85E-02*	0.65	5.31E-01	0.04
7	Pec	pQTL	47	rs3676254	rs3656205	*5.61E-03*	1.25	*2.84E-03*	1.16	*3.39E-03*	0.85
9	Fat	pQTL	15	rs3719607	rs8259427	*2.31E-02*	0.95	*1.02E-02*	0.92	4.53E-01	0.06
9	AI	pQTL	15	rs3719607	rs8259427	*3.50E-02*	0.85	*1.61E-02*	0.82	5.38E-01	0.04
11	AI	pQTL	14	rs6276300	rs6199956	*3.29E-02*	0.87	*4.53E-02*	0.62	*4.35E-02*	0.41
11	Fat	pQTL	15	rs6276300	rs6199956	*3.75E-02*	0.85	*4.80E-02*	0.61	*3.65E-02*	0.44
11	Gastro	pQTL	24	rs6276300	rs6199956	*3.00E-04*	1.90	*1.77E-04*	1.74	*3.23E-05*	1.74
11	BMI	pQTL	49	rs13481054	rs3701609	1.44E-01	0.54	6.64E-02	0.54	*2.85E-02*	0.48
14	BMI	pQTL	65	rs3709178	rs13482404	*1.54E-02*	1.03	1.16E-01	0.43	5.64E-01	0.03
17	Gastro	pQTL	11	rs13482893	rs3719497	1.15E-01	0.59	5.29E-02	0.59	*1.67E-02*	0.57
17	AI	pQTL	13	rs13482893	rs3719497	5.86E-02	0.73	*2.56E-02*	0.72	*4.15E-02*	0.41
17	Fat	pQTL	13	rs13482893	rs3719498	*4.83E-02*	0.78	*2.11E-02*	0.77	*4.19E-02*	0.41

^a^Trait abbreviations are the same as in Table 1. P: total protein.

^b^Peak position of QTL detected in Kosambi centimorgans.

^c^Flanking markers (left and right) of the QTL peak. See Additional file 1: Table S6 for marker information.

^d^am + dm + im tested the overall interaction, which included additive, dominance, and imprinted pQTL by sex interactions. *P*-value < 0.05 is shown in italics %var: percentage of phenotypic variance accounted for at QTL position.

^e^am + dm tested for non-imprinted interactions, which included additive and dominance pQTL by sex interactions. *P*-value < 0.05 is shown in italics %var: percentage of phenotypic variance accounted for at QTL position.

^f^am tested the additive interaction, which included additive pQTL by sex interactions. *P*-value < 0.05 is shown in italics %var: percentage of phenotypic variance accounted for at QTL position.

**Table 5:** Statistics of QTL that interact with reciprocal cross

			**Position**	**Flanking markers**^**c**^		**am + dm + im**^**d**^		**am + dm**^**e**^		**am**^**f**^	
**Chr**	**Trait**^**a**^	**Groups**	**(cM)**^**b**^	**Left**	**Right**	***P*-value**	**% var**	***P*-value**	**% var**	***P*-value**	**% var**
1	Gastro	pQTL	23	rs3696088	rs13472794	*2.85E-06*	2.68	*4.41E-06*	2.32	*3.14E-06*	2.06
1	Pec	pQTL	23	rs3696088	rs13472794	*1.62E-02*	0.94	7.04E-02	0.49	*5.78E-03*	0.71
1	Tnni1	eQTL	24	rs3696088	rs13472794	*3.03E-02*	1.14	*1.25E-02*	1.12	*4.84E-03*	1.02
1	Edl	pQTL	25	rs3696088	rs13472794	*4.02E-04*	1.81	*8.59E-03*	0.95	*4.14E-03*	0.82
1	R	tQTL	29	rs13472794	rs13475931	*1.09E-05*	3.50	*2.03E-02*	1.09	1.10E-01	0.36
1	R/D	tQTL	29	rs13472794	rs13475931	*1.39E-04*	2.82	*1.81E-02*	1.12	1.65E-01	0.27
6	lengthNA	pQTL	63	mCV24115224	UT_6_123.37228	*3.24E-02*	0.87	5.46E-02	0.58	*2.81E-02*	0.48
7	Pec	pQTL	47	rs3676254	rs3656205	*3.82E-02*	0.84	*1.64E-02*	0.82	1.25E-01	0.24
7	IGF1	eQTL	53	rs13479422	rs13479471	*1.62E-02*	1.33	*6.47E-03*	1.31	*5.18E-03*	1.02
7	Tail	pQTL	61	rs13479471	rs6275579	*3.45E-02*	0.86	5.23E-01	0.13	6.42E-01	0.02
11	Fat	pQTL	57	rs3701609	rs8270290	5.27E-02	0.77	*1.76E-02*	0.81	8.18E-02	0.31
11	AI	pQTL	57	rs3701609	rs8270290	6.46E-02	0.72	*2.18E-02*	0.76	1.06E-01	0.26
13	R	tQTL	25	rs13481780	rs3678784	*1.08E-03*	2.21	*2.08E-04*	2.34	*1.26E-03*	1.44
13	R/D	tQTL	25	rs13481780	rs3678784	*2.05E-03*	2.02	*4.71E-04*	2.12	*4.36E-03*	1.13
14	R/D	tQTL	18	rs13482096	rs8251329	*3.64E-04*	2.56	*6.77E-04*	2.03	*1.05E-03*	1.50
17	Edl	pQTL	31	rs3023442	rs6395919	2.00E-01	0.47	9.81E-02	0.47	*3.46E-02*	0.45

^a^Trait abbreviations are the same as in Table 1. R: total RNA; R/D: total RNA/total DNA.

^b^Peak position of QTL detected in Kosambi centimorgans.

^c^Flanking markers (left and right) of the QTL peak. See Additional file 1: Table S6 for marker information.

^d^am + dm + im tested the overall interaction, which included additive, dominance, and imprinted pQTL by cross interactions. *P*-value < 0.05 is shown in italics % var: percentage of phenotypic variance accounted for at QTL position.

^e^am + dm tested for non-imprinted interactions, which included additive and dominance pQTL by cross interactions. *P*-value < 0.05 is shown in italics % var: percentage of phenotypic variance accounted for at QTL position.

^f^am tested for additive interactions, which included additive QTL by cross interactions. *P*-value < 0.05 is shown in italics %var: percentage of phenotypic variance accounted for at QTL position.

Significant interactions with *Myostatin* genotype, sex, and reciprocal cross were also detected for expression traits (Tables 3, 4 and 5 respectively). These eQTL were located on chromosomes 1, 3, 6, 7, 8, and X. Similar to the pQTL data, the *P*-values from three interaction tests are presented, along with the phenotypic variation explained by these interaction models.

Using a comparison-wise *P*-value of less than 0.05, a total of seven tQTL were identified for their significant interaction with *Myostatin* genotype, sex, or reciprocal cross (Tables 3, 4 and 5). Among these seven tQTL, five of them interacted with reciprocal cross, one with *Myostatin* genotype, and one with sex. The average variation accounted for by these QTL was about 2.5%.

### Genetic variation components

**Figure 2:**
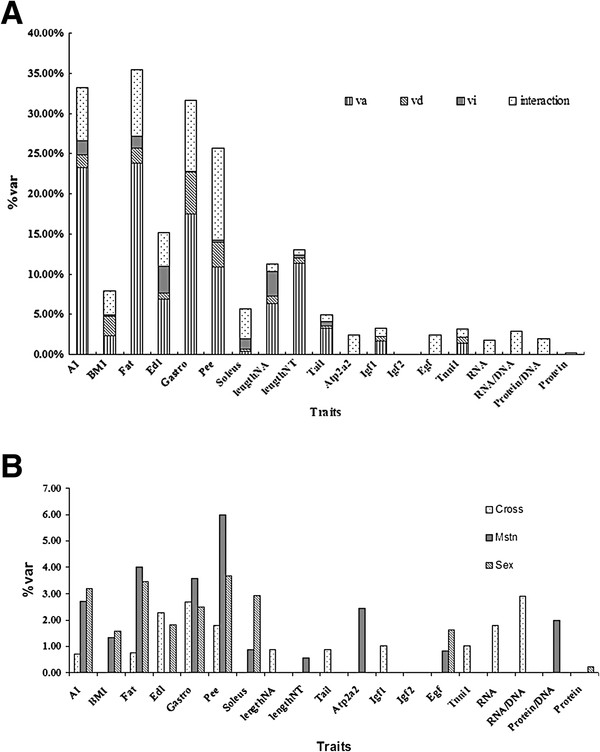
**Proportion of phenotypic variation accounted for by identified QTL. (A)** Phenotypic variation accounted for by additive, dominance and imprinted QTL effects. interaction: the sum of *Myostatin* genotype by QTL, reciprocal cross by QTL, and sex by QTL effect. vi: imprinted QTL effect. vd: dominance QTL effect. va: additive QTL effect**. (B)**. Phenotypic variation accounted for by QTL that interacted with *Myostatin* genotype, reciprocal cross, and sex. Trait abbreviations are the same as in Table 1. Mstn: *Myostatin* genotype by QTL interaction. Cross: Reciprocal cross by QTL interaction. Sex: Sex by QTL interaction.

For each trait, the total amount of phenotypic variation accounted for by additive, dominance, and imprinted QTL is presented in Figure 2(A). For most traits, the largest proportion of phenotypic variation could be accounted for by additive and dominance QTL. Additive, dominance, and interaction QTL effects explained almost equal amounts of genotypic variation for BMI. In contrast, QTL interactions could account for a large proportion of the phenotypic variation in soleus weight. The amount of phenotypic variation explained by imprinted QTL varied from trait to trait and was relatively small for most traits. In comparison to pQTL, the amount of phenotypic variation accounted for by eQTL and tQTL was relatively small.

The amount of phenotypic variation accounted for by interactions is summarized in Figure 2(B). For fat-related traits, pQTL by *Myostatin* genotype or sex interactions explained the majority of the phenotypic variation. In contrast, pQTL by cross interactions explained more of the phenotypic variation in muscle weight traits. Interestingly, no pQTL interactions were identified for body length traits. Overall, the amount of phenotypic variation that could be accounted for by QTL interactions was very small for tQTL and eQTL traits.

## Discussion

### Imprinting effects on body size and adipose traits

We identified six imprinted QTL. The reason we were able to detect these imprinted QTL was because the two mouse lines used in this study were not fully inbred. In mice, a few imprinted QTL have been previously identified. For example, Leamy et al. [41] used a post hoc t-test [6] from regression analyses and discovered several QTL that displayed an imprinted inheritance pattern for mandible size and shape in mice. These QTL were located on chromosomes 2, 3, 6, and 12. Imprinted QTL have also been identified on mouse chromosome 8 for a mature body mass trait [42]. In addition, there was evidence to support the possibility that some imprinted genomic regions on mouse chromosomes 3, 4, 5, 6, 7, 12, 18 and 19 had effects on adult body composition and muscle traits [43-45]. Based on these previous mapping results, chromosomes 10 and 11 have not been previously shown to harbor QTL that influence body length traits.

The imprinted QTL identified on chromosome 18 was associated with fat-related traits. This region has not been previously identified as potentially harboring imprinted QTL in other studies, likely due to the limited amount of research conducted to identify imprinted QTL that influence adipose accumulation. In mice, potential imprinted obesity QTL were first identified in LGXSM recombinant inbred strains [46]. Although this imprinting effect may confound with maternal effect. Other studies provide additional support for the presence of imprinted QTL on chromosomes 2 and 7 that are associated with fat pad weight in mice [47]. Imprinted obesity QTL in other species, such as humans and pigs [48-50], also indicate that imprinted QTL can account for significant amounts of the variation observed in muscle mass and fat deposition traits.

### pQTL control of muscle and adipose traits

Using interval mapping and the genome-wide permutation method, we identified a number of additive and dominance pQTL that were associated with muscle weight and fat-related traits. This is understandable given the mouse lines used in this study. The most significant phenotypic differences observed between M16i and C57BL/6 *Myostatin*-null lines were in skeletal muscle weight and fat accumulation. We expected that loci associated with these phenotypes would segregate in the F2 generation and could be identified through pQTL mapping. Most of the estimated QTL effects were small. These results support our current understanding of genomic architecture, in that quantitative traits are controlled by numerous genes each with small effects, as well as a few genes with large effects.

The chromosomes that were associated with significant pQTL effects contained some promising candidate genes for muscle, adipose, and body size development. For example, IGF-binding protein 2 (*Igfbp2*), located at 36 cM on chromosome 1, has been shown to modulate IGF1 activity and thereby protect against obesity [51]. This is in close proximity to our fat QTL at 24 cM on chromosome 1. In close proximity to *Igfbp2*, IGF-binding protein 5 (*Igfbp5*) on chromosome 1 is another candidate gene which is known to impact whole-body growth and muscle development [52]. On chromosome 7, the insulin-like growth factor 1 receptor gene (*IGF1r*) at 33 cM could be the gene underlying our muscle QTL at 47 cM on the same chromosome. The growth hormone gene (Gh) at 65 cM on chromosome 11is located close to the position of our gastrocnemius QTL at 68 cM. Variants in these genes have been associated with overgrowth [53], obesity [54] and insulin resistance [55], which could have more widespread effects for other tissues, e.g. skeletal muscle growth.

Some of the pQTL that were associated with AI and fat weight overlapped with one another (see Table 1). This finding is not unexpected, given the high positive phenotypic correlation between these two traits. However, the pQTL identified on chromosome 11 for BMI was not associated with either AI or fat weight. pQTL for fat-related traits (e.g., body fat mass and body mass) have been mapped to this region previously [56-58]. The fact that BMI, AI, and fat weight pQTL were not identical supports the importance of using multiple measurements of obesity. BMI was first described in the 19^th^century and has been widely used in clinical obesity research. BMI takes into account body size information that might not be elucidated by AI and fat weight measurement alone. Identification of genetic variants that are associated with BMI at different growth periods should help to understand the genetic mechanisms that underlie BMI [57,59-61].

### Inheritance pattern of QTL that interact with *Myostatin* genotype, reciprocal cross and sex

We tested the identified pQTL for possible interactions with *Myostatin* genotype, reciprocal cross, and sex. In addition, we evaluated the nature of the inheritance pattern of these pQTL interactions (i.e., additive, dominance, or imprinted) by comparison of different QTL models. For example, many of the QTL that interacted with *Myostatin* genotype, reciprocal cross, and sex appeared to be inherited in either an additive (e.g., the gastrocnemius weight pQTL on chromosome 17 that interacted with sex; Table 4) or dominant (e.g., the fat pad weight pQTL on chromosome 11 that interacted with reciprocal cross; Table 5) fashion. Meanwhile, there were other pQTL that did not have a significant additive interaction effect (am) or combination interaction effect (am + dm), but that were potentially inherited in an imprinted manner (e.g., the EDL weight pQTL on chromosome 1 that interacted with sex; Table 4). These statistical testing results indicate that the interaction pattern between a given QTL and *Myostatin* genotype, cross, or sex is complicated, and further molecular experiments with these loci will be needed in order to elucidate the inheritance pattern.

We estimated the phenotypic variation accounted for by the identified QTL. Compared to additive and dominant QTL effects, individual interactions generally explained a small proportion of the total phenotypic variation (<2% of the total). In our previous pQTL mapping study [12], we discovered a number of QTL interactions that also explained a small amount of phenotypic variation in growth traits. These results indicate that, for quantitative traits, the amount of variation explained by gene-gene interactions appears to be smaller than for additive and dominant QTL. However, in this study, we only evaluated QTL by *Myostatin* genotype, which is a small proportion of the possible gene-gene interactions. Thus, the total amount of phenotypic variation explained by these gene-gene interactions could still be large if all possible gene-gene interactions within the genome were accounted for.

### Integration of tQTL, eQTL, and pQTL information

Three tQTL on chromosomes 1, 13, and 14 significantly interacted with cross to affect the total RNA amount and RNA/DNA ratio in the pectoralis. RNA/DNA ratio is a rough measure of transcriptional rate. Therefore, it would appear that there are some alleles that segregate between these two lines that control overall transcription level. Two other tQTL on chromosome 2 were associated with total protein amount and the protein/DNA ratio (Tables 3 and 4). These two tQTL significantly interacted with *Myostatin* genotype and sex. The amount of protein and protein/DNA ratio in a tissue are indicators of muscle hypertrophy, which is known to result when *Myostatin* is postnatally inactivated in mice [62]. Therefore, these two QTL could be key mediators of protein accretion that are controlled by *Myostatin* and sex to regulate muscle hypertrophy.

It has been demonstrated that IGF1 regulates skeletal muscle growth by promotion of satellite cell proliferation and muscle protein synthesis [63,64]. Previously, we reported that the genomic region surrounding 65 cM on chromosome 4 was significantly associated with average daily gain (from the 1st to 3rd week) and body weight (the 3rd, 4th, and 5th week) traits (Cheng et al., 2011). Here, we identified an eQTL for *IGF1* expression at 68 cM on chromosome 4. Considering that the *IGF1* gene is located on mouse chromosome 10, these results indicate that it is highly possible that a trans-regulatory element of *IGF1* expression is located on chromosome 4. This element might control the expression level of *IGF1* to further regulate body growth. In addition, another *IGF1* eQTL was identified on chromosome 7 that significantly interacted with reciprocal cross (Table 5). In addition, we detected muscle pQTL that also interacted with cross and sex near this position (Tables 4 and 5). These results support the possibility that in addition to genetic background, IGF1 contributes to the sexual dimorphism in whole body weight and muscle growth, likely via GH and the Stat5b signaling pathway [65,66].

We evaluated the positional concordance between pQTL and eQTL. Amongst them, we observed a remarkable co-localization of pectoralis and gastrocnemius weight pQTLs on chromosome 1 (23 cM) and a *cis-*acting eQTL regulating the expression levels of *Tnni1*. *Tnni1* gene is associated with tropomyosin and regulates the calcium sensitivity of the myofibril contractile apparatus of striated muscles.

We identified two eQTL that interacted with *Myostatin* genotype to control *Atp2a2* expression level. These two eQTL were not located on the same chromosome as *Atp2a2* and might be long-distance regulatory factors. Recently, it was shown that a change in *Atp2a2*expression level was representative of a fiber-type transformation [67]. In addition, proteomic analysis of skeletal muscle in cattle demonstrated that *Myostatin* genotype impacted muscle fiber composition [68]. Thus, it is possible that *Myostatin* might interact with these two eQTL to regulate the *Atp2a2* activity, thereby altering fiber type.

## Conclusions

Imprinted pQTL were identified on chromosomes 6, 9, 10, 11, and 18 that were associated with muscle weight, fat-related, and body length traits. Furthermore, pQTL, tQTL, and eQTL that interacted with *Myostatin* genotype, reciprocal cross, and sex were identified across the genome. These results indicate that gene-gene interactions are widely involved in muscle and adipose development.

## Methods

### Mouse lines and breeding procedure

Two founder mouse strains, *Myostatin*-null C57BL/6 [69] and M16i high body weight [34], were reciprocally crossed to derive an F2 mapping population. The *Myostatin*-null C57BL/6 line contained a non-functional *Myostatin* gene on both chromosomes. The M16i obese mouse line was derived from an outbred population (ICR) for high weight gain prior to six weeks of age. To generate the F2 progeny, four *Myostatin*-null male mice were crossed with eight M16i females. This generated 35 male and 37 female F1 mice. Meanwhile, two M16i males and seven *Myostatin*-null females were mated in the reciprocal cross to produce 31 male and 55 female F1 offspring. The resulting F1 mice were intercrossed within each reciprocal cross to obtain the final F2 mapping population.

### Trait collection procedures

One day after birth, each F2 litter was standardized to nine pups. Mice were identified by toe clipping seven days after birth. At three weeks of age, mice were weaned and placed in separate cages. We obtained 552 mice from the *Myostatin*-null male by M16i female cross and 448 mice from the reciprocal cross. These 1,000 F2 mice were either homozygous *Myostatin* wild-type or homozygous *Myostatin*-null genotype. At 42 days of age, body weight was recorded for each F2 individual. Subsequently, mice were sacrificed. Skeletal muscles (soleus, gastrocnemius, EDL, and pectoralis) from both the left and right sides of the body, as well as gonadal fat pads (epididymal for males and perimetrial for females) were collected and weighed. The tissue weight percentage was calculated as the percentage of tissue weight divided by the 6th week body weight. Body size traits (nasal-tail length, nasal-anal length, and tail length) were also measured. Based on these measurements, body mass index (BMI) and adiposity index (AI) were calculated and included as measurements of obesity [70]: BMI = 6th week body weight / square of the anal-nasal length; AI = 6th week body weight / fat weight. All weights were measured in grams (g), while all lengths were measured in centimeters (cm).

A proportion of the right pectoralis muscle of each F2 mouse was used for total RNA, DNA, and protein isolation using a standard protocol [71]. For tQTL mapping, the ratios of DNA/RNA, protein/RNA and protein/DNA were calculated based on these measurements. The remainder of the muscle was homogenized in liquid nitrogen. After homogenization, total RNA was isolated using RNeasy (Qiagen). The resulting RNA was amplified and measured by a triplex qPCR using Quantitect (Qiagen). Three sets of triplex qPCR assays were performed. The first set included beta-actin (Actb), adipophilin (Adfp) and ATPase2 (Atp2a2). The second set included epidermal growth factor (Egf), insulin-like growth factor 1 (IGF1) and insulin-like growth factor 2 (Igf2). The third set included myogenic factor 5 (Myf5), troponinI (Tnni1) and wingless-related MMTV integration site 4 (Wnt4). qPCR primers are listed in Additional file 1: Table S5. Each sample was measured twice, and the average CT value was then normalized by the CT value of Actb and by the plate efficiency. The adjusted CT values were then used as traits for eQTL mapping. All animal procedures were approved by the Iowa State University Animal Care and Use Committee prior to this study.

### Genotyping and linkage map

Genomic DNA was isolated from toe clips and purified by a phenol chloroform method. A total of 242 SNPs evenly spaced across19 autosomes and the X chromosome were genotyped on the 1,000 F2 mice, in addition to the *Myostatin* locus. The *Myostatin* locus was genotyped by standard PCR and agarose gel electrophoresis protocols. The SNP genotyping procedure was performed on the Sequenom® platform at GeneSeek® (Lincoln, Nebraska). SNPs on chromosome 15 and chromosome 16 were discarded because no informative SNPs were present. Among the remaining SNPs, 152 SNPs with call rates greater than 80% and no observable genotyping errors were included in the analysis. These 152 SNPs were located on 17 autosomes and the X chromosome. Marker segregation distortion was evaluated in the F2 mice by a chi-square test. Only SNPs close to the *Myostatin* locus significantly deviated from the expected Mendelian segregation ratios. This was caused by the fact that only homozygotes at the *Myostatin* locus were included. All 152 SNP markers were used to generate a linkage map by CRIMAP [72], with distances estimated in Kosambi centimorgans. The marker order and position in our map (Additional file 1: Table S6) were consistent with the map from the Wellcome-CTC Mouse Strain SNP Genotype Set. Therefore, we analyzed the data based on our linkage map.

### Data analyses

#### Data exploration

Simple statistics (mean, standard deviation, minimum and maximum) were calculated for each trait. In addition, all main factors (*Myostatin* genotype, sex, reciprocal cross, coat color) and interaction terms (interactions between main effects) were tested for each trait by fitting a generalized linear model. Effects with a *P*-value of less than 0.1 were included in the QTL model (Additional file 1: Table S3). Significant factors were removed from the linear model to evaluate the residual correlations between each pair of trait values. All general statistical analyses of the F2 data were carried out using a SAS® software package. The corresponding procedures used were PROCMEANS, PROCGLM and PROCCORR.

#### Additive, dominance, and imprinting effects

First, we analyzed each trait to identify imprinted QTL. To achieve this, two different models were used to perform a whole-genome scan. The imprinted QTL model included significant main effects and interaction effects, along with additive, dominance, and parent-of-origin (imprinting) effects at a single QTL position. The non-imprinted QTL model included all the terms in the first model except for the imprinting effect. Each QTL model was individually analyzed in GridQTL [73], a web-based QTL analysis program to identify QTL by interval mapping. A genome-wide permutation procedure [74] with 1,000 repetitions was applied to each model to obtain 1% and 5% genome-wide significance levels. All QTL positions above the 5% significance level under the imprinted QTL model and the non-imprinted QTL model were used to evaluate the imprinting effect by calculating an F-value as follows:

d.f. : degree of freedom of error term (same after)

SSE : sum squares of error

The corresponding comparison-wise *P*-value was computed from a standard F-distribution with the corresponding degrees of freedom. If the *P*-value was less than 0.05, the imprinted QTL model was assumed to be more suitable for this QTL position. Otherwise, the non-imprinted model was chosen. After the best model for each trait was determined, it was applied to each QTL position again to obtain F-values, LOD scores and estimates for QTL effects, along with the corresponding standard errors for each QTL peak.

At each QTL position, a corresponding comparison-wise *P*-value was computed for the additive and dominance effects respectively, as follows:

Additive and dominance model (AD model):

$$\begin{array}{l} \text{Phenotypic}\;\text{value}\;=\;\text{fixed}\;\text{effects}\;+\;\text{additive}\;\text{effect}\\ \;\times \;\left(\text{QTL}\;\text{position}\right)\\ \;+\text{dominance}\;\text{effect}\;\left(\text{QTL}\;\text{position}\right)+\epsilon \end{array}$$

Additive model (A model):

$$\begin{array}{l} \text{Phenotypic}\;\text{value}=\text{fixed}\;\text{effects}+\text{additive}\;\text{effect}\\ \;\times \;\left(\text{QTL}\;\text{position}\right)+\epsilon \end{array}$$

Full reduced model (R model):

Phenotypicvalue=fixedeffects+ϵ

For additive effect:

$$F=\frac{\text{SSE}\;\left(R\;\text{model}\right)‒\text{SSE}\;\left(A\;\text{model}\right)}{\text{SSE}\;\left(A\;\text{model}\right)/d.f.\left(A\;\text{model}\right)}$$

For dominance effect:

$$F=\frac{\text{SSE}\;\left(A\;\text{model}\right)‒\text{SSE}\;\left(\text{AD}\;\text{model}\right)}{\text{SSE}\;\left(\text{AD}\;\text{model}\right)/d.f.\left(\text{AD}\;\text{model}\right)}$$

We calculated *P*-values from an F-distribution with the corresponding degrees of freedom. We named this *P*-value as a comparison-wise *P*-value.

The phenotypic variance explained by the QTL was computed. For imprinted QTL, the percentage of phenotypic variation accounted for by a QTL position was computed as the percentage of residual sum of squares explained by the additive, dominance, and imprinting effects at the QTL using the imprinted QTL model. For non-imprinted QTL, the percentage of phenotypic variation accounted for by a QTL position was computed as the percentage of residual sum of squares explained by the additive and dominance effects using the non-imprinted QTL model.

#### QTL interaction analysis

We first analyzed the interaction between QTL position and *Myostatin* genotype. To accomplish this, we split the data into two subsets based on *Myostatin* genotype. Within each *Myostatin* genotype subset, both the imprinted QTL model and the non-imprinted QTL model were evaluated by interval mapping. All QTL positions identified at 5% genome-wide significance levels in either model were considered for analysis in the next step, in which the following six QTL models were fitted to these identified QTL positions in the full F2 dataset.

Model 1–1:

$$\begin{array}{l} \text{Phenotypic}\;\text{value}=\text{fixed}\;\mathrm{effects}+\mathrm{additive}\;\mathrm{effect}\\ \;\times \;\left(\mathrm{QTL}\;\mathrm{position}\right)+\mathrm{dominance}\;\mathrm{effect}\\ \;\times \;(\mathrm{QTL}\;\mathrm{position})+\mathrm{imprinting}\;\mathrm{effect}\\ \;\times \;\left(\mathrm{QTL}\;\mathrm{position}\right)+\;\text{Myostatin}\times \;\text{additive}\;\text{effect}\\ \;\times \;(\text{QTL}\;\text{position})\\ \;+\text{Myostatin}\times \mathrm{dominance}\;\text{effect}\\ \;\times \;\left(\text{QTL}\;\text{position}\right)+\text{Myostatin}\\ \;\times \text{imprinting}\;\text{effect}\;(\text{QTL}\;\text{position})+\epsilon \end{array}$$

Model 1–2:

$$\begin{array}{l} \text{Phenotypic}\;\text{value}=\text{fixed}\;\text{effects}+\text{additive}\;\text{effect}\\ \;\times \;\left(\text{QTL}\;\text{position}\right)+\text{dominance}\;\mathrm{effect}\\ \;\times \;(\mathrm{QTL}\;\mathrm{position})+\text{imprinting}\;\text{effect}\\ \;\times \;\left(\text{QTL}\;\text{position}\right)+\epsilon \end{array}$$

Model 2–1:

$$\begin{array}{l} \text{Phenotypic}\;\text{value}\;=\;\text{fixed}\;\text{effects}+\text{additive}\;\text{effect}\\ \;\times \;\left(\text{QTL}\;\text{position}\right)\;+\;\text{dominance}\;\text{effect}\\ \;\times \;(\text{QTL}\;\text{position})+\text{Myostatin}\\ \;\times \;\text{additive}\;\text{effect}\;\left(\text{QTL}\;\text{position}\right)\\ \;+\text{Myostatin}\times \text{dominance}\;\text{effect}\\ \;\times \;(\text{QTL}\;\text{position})+\epsilon \end{array}$$

Model 2–2:

$$\begin{array}{l} \text{Phenotypic}\;\text{value}\;=\;\text{fixed}\;\text{effects}+\text{additive}\;\text{effect}\\ \;\times \;\left(\text{QTL}\;\text{position}\right)\\ \;+\text{dominance}\;\text{effect}\;\left(\text{QTL}\;\text{position}\right)\\ \;+\epsilon \end{array}$$

Model 3–1:

$$\begin{array}{l} \text{Phenotypic}\;\text{value}\;=\;\text{fixed}\;\text{effects}\;+\;\text{additive}\;\text{effect}\\ \;\times \;\left(\text{QTL}\;\text{position}\right)+\;\text{Myostatin}\\ \;\times \text{additive}\;\text{effect}\;\left(\text{QTL}\;\text{position}\right)+\epsilon \end{array}$$

Model 3–2:

$$\begin{array}{l} \text{Phenotypic}\;\text{value}\;=\;\text{fixed}\;\text{effects}\;+\;\text{additive}\;\text{effect}\\ \;\times \;\left(\text{QTL}\;\text{position}\right)+\epsilon \end{array}$$

By comparing the above models, we determined the F-values for different interaction effects as follows:

1) *When considering the general interaction (additive, dominance and imprinting interaction):*

2) *When considering the non-imprinting interaction (additive and dominance interaction):*

3) *When considering the additive interaction:*

Correspondingly, each F-value gave a comparison-wise *P*-value. Interactions with a *P*-value of less than 0.05 were considered significant.

Using a similar approach, reciprocal cross × QTL interactions were analyzed. Initially, the data was split into two subsets by reciprocal crosses and potential QTL positions for testing. Reciprocal cross interactions were identified within each cross subset. Then, interactions at each position were tested in the full dataset, similar to the procedure used for *Myostatin* interactions, but with the *Myostatin* effect replaced by the reciprocal cross effect in models 1–1 to 3–2.

Finally, the same approach was applied to identify sex × QTL interactions. The data was split into two subsets by sex and potential QTL positions were identified within each sex subset. *Myostatin* effect in the QTL models was replaced by sex effect, and the corresponding interaction effects were switched, as well.

Estimates of additive and dominance effects and phenotypic variation accounted for by each identified QTL were estimated in the same way discussed above for main effect QTL. To calculate the total effect of pQTL, we summed the phenotypic variation explained by all main effect pQTL and interaction pQTL, for each trait respectively.

## Abbreviations

QTL: Quantitative trait loci; pQTL: Phenotypic quantitative trait loci; tQTL: Translation and transcription quantitative trait loci; eQTL: Expression quantitative trait loci; AI: Adiposity index; FAT: Fat pad weight percentage; BMI: Body mass index.

## Competing interests

The author(s) declare that they have no competing interests.

## Authors’ contributions

YC and SR participated in the design of the study, carried out the mice tissue studies and drafted the manuscript. YC and AC carried out the gene expression measurement. MSM and RJT MT helped the mice collection. YC performed the statistical analysis. JMR conceived of the study, and participated in its design and coordination and helped to draft the manuscript. JCMD participated in the study design and statistical analysis. All authors read and approved the final manuscript.

## Supplementary Material

Additional file 1 Includes Table S1 to Table S6, which give detail information about the statistical summary of traits measured in this study, the estimated effects of identified QTLs and SNP markers. Click here for file

Additional file 2 Includes Table S7, which gives detail information about pairwise correlation among all traits measured in this study. Click here for file
