# Supplementary material for: Body composition and gene expression QTL mapping in mice reveals imprinting and interaction effects
Source: BMC Genet. 2013 Oct 29;14:103. doi: 10.1186/1471-2156-14-103 (PMC4233306; doi:10.1186/1471-2156-14-103)
Supplement: Additional file 1 — Includes Table S1 to Table S6, which give detail information about the statistical summary of traits measured in this study, the estimated effects of identified QTLs and SNP markers. [file 1471-2156-14-103-S1.doc]

**Supplementary information**

**Supplemental Table 1. Basic statistics for phenotypic traits measured in the F2 mapping population**

| Groups | Traits^a^ | Mean | Std. Dev^b^ | Range^c^ |
| --- | --- | --- | --- | --- |
| Phenotype | lengthNA | 9.926 | 0.728 | 7.000-16.700 |
|  | lengthNT | 17.691 | 1.203 | 12.000-20.500 |
|  | AI | 0.470 | 0.250 | 0.041-1.743 |
|  | BMI | 30.598 | 4.312 | 10.517-45.793 |
|  | Tail | 7.764 | 0.784 | 2.100-10.000 |
|  | Soleus | 0.032 | 0.015 | 0.010-0.381 |
|  | Gastro | 0.592 | 0.113 | 0.367-1.013 |
|  | Edl | 0.358 | 0.080 | 0.193-1.649 |
|  | Pec | 0.636 | 0.245 | 0.277-1.310 |
|  | Fat | 0.470 | 0.250 | 0.041-1.743 |
| Translation | D | 7.292 | 1.355 | 2.138-13.820 |
|  | R | 1.497 | 0.819 | 0.101-13.980 |
|  | P | 0.602 | 0.200 | 0.149-2.450 |
|  | R/D | 0.205 | 0.086 | 0.019-1.225 |
|  | P/R | 0.468 | 0.243 | 0.043-4.864 |
|  | P/D | 0.088 | 0.027 | 0.019-0.341 |
| Expression | Adfp | 2.409 | 2.735 | -14.280-8.414 |
|  | Atp2a2 | 3.652 | 7.756 | -4.178-43.460 |
|  | EGF | 3.820 | 4.754 | -9.364-28.190 |
|  | Igf1 | 1.870 | 1.294 | -1.154-7.439 |
|  | Igf2 | -0.444 | 2.730 | -17.790-5.423 |
|  | Myf5 | 2.649 | 6.970 | -8.232-29.670 |
|  | Tnni1 | 2.441 | 1.980 | -5.292-7.322 |
|  | Wnt4 | 5.094 |  | -3.389-73.950 |

^a^ lengthNA: nasal to anal length (cm); lengthNT: nasal to tail length (cm); AI: adiposity index; BMI: body mass index; Tail: tail length (cm); Soleus: soleus muscle weight percentage; Gastro: gastrocnemius muscle weight percentage; Edl: EDL muscle weight percentage; Pec: pectoralis muscle weight percentage; Fat: average gonadal fat pad weight percentage (epididymal for males and perimetrial for females); D: DNA; R: RNA; P: protein; R/D: RNA/DNA; P/R: protein/RNA; P/D: protein/DNA.

^b^ standard deviation

^c^ minimum and maximum

**Supplemental Table 2. Phenotypic correlation among phenotypic traits^a^**

| Traits^b^ | lengthNA | lengthNT | AI | BMI | Tail | Soleus | Gastro | Edl | Pec | Fat |
| --- | --- | --- | --- | --- | --- | --- | --- | --- | --- | --- |
| lengthNA | 1.0 | 0.778** | 0.308** | 0.013* | 0.264** | -0.004* | 0.112 | -0.051* | 0.097 | 0.309** |
| lengthNT | - | 1.0 | 0.377** | 0.158** | 0.812** | -0.043* | 0.020* | -0.111 | -0.014* | 0.377** |
| AI | - | - | 1.0 | 0.008* | 0.291** | -0.016** | -0.454** | -0.439** | -0.382** | 1.000** |
| BMI | - | - | - | 1.0 | 0.231** | 0.110 | 0.550** | 0.347** | 0.676** | 0.008* |
| Tail | - | - | - | - | 1.0 | -0.063 | -0.073 | -0.123** | -0.111 | 0.291** |
| Soleus | - | - | - | - | - | 1.0 | 0.351** | 0.301** | 0.290** | -0.164** |
| Gastro | - | - | - | - | - | - | 1.0 | 0.668** | 0.886** | -0.454** |
| Edl | - | - | - | - | - | - | - | 1.0 | 0.606** | -0.439** |
| Pec | - | - | - | - | - | - | - | - | 1.0 | -0.382** |
| Fat | - | - | - | - | - | - | - | - | - | 1.0 |

^a^ P-value for testing for significant correlation. **: P < 0.0001; *: 0.0001 < P < 0.05; otherwise, P > 0.05. Phenotypic correlation among all traits are included in corr.xls file.

^b^ Trait abbreviations are the same as in Supplemental Table 1.

**Supplemental Table 3. Effects included in the QTL model for each trait analyzed^a^**

| Effects | Muscle | AI | BMI | Body length | Transcription and translation | Gene expression |
| --- | --- | --- | --- | --- | --- | --- |
| *Myostatin* genotype (M) | * | * | * | * | * | * |
| Sex (S) | * | * | * | * | * | * |
| Reciprocal cross (R) | * | * | * | * | * | * |
| Coat color (C) | * | * | * | * | * | * |
| M * S | * |  |  |  | * | * |
| M * R | * |  |  |  | * | * |
| M * C |  |  |  |  |  |  |
| S * R | * |  |  |  |  | * |
| S * C | * |  |  |  |  |  |
| R * C | * |  |  |  | * | * |
| M * S * R | * | * | * | * |  | * |
| M * S * C | * | * | * | * |  |  |
| M * R * C | * |  |  |  | * |  |
| S * R * C | * |  |  |  |  | * |
| M * S * R *C | * |  |  |  |  | * |
| Group |  |  |  |  | * |  |
| Plate |  |  |  |  |  | * |
| Replicate |  |  |  |  |  | * |

^a^ Effects in the QTL model. M: *Myostatin* genotype; S: sex; R: reciprocal cross; C: coat color; Group: group effect in DNA, RNA and protein isolation; Plate: plate effect in real time PCR experiment; Replicate: replicate effect in real time PCR experiment. A notation of “ *” indicates that the corresponding effect is included in the QTL model. Blank indicates that the corresponding effect is not included in the QTL model. Traits are grouped into: muscle (soleus, gastrocnemius, EDL, pectoralis); AI (adiposity index); BMI (body mass index); body length (nasal to anal length, nasal to tail length and tail length); transcription and translation (total RNA, total DNA, total protein, DNA/RNA, RNA/protein and DNA/protein); gene expression (Actb, Adfp, Atp2a2, Egf, IGF1, Igf2, Myf5, Tnni1 and Wnt4).

**Supplemental Table 4. Test statistics of additive, dominance and imprinted QTL**

|  |  | Peak^b^ | *P*-value^c^ | | |
| --- | --- | --- | --- | --- | --- |
| MMU | Trait^a^ | (cM) | i | a | d |
| 1 | Gastro | 23 | - | **2.84E-14** | **1.16E-03** |
| 1 | Pec | 23 | - | **3.22E-20** | **3.10E-05** |
| 1 | Tnni1 | 23 | - | **2.96E-04** | **4.89E-02** |
| 1 | AI | 24 | - | **8.79E-07** | 1.30E-01 |
| 1 | Fat | 24 | - | **1.01E-06** | 9.83E-02 |
| 1 | Edl | 25 | - | **1.58E-04** | 9.18E-01 |
| 2 | Edl | 78 | - | **5.92E-04** | 1.94E-01 |
| 2 | Gastro | 80 | - | **3.05E-04** | 1.82E-02 |
| 2 | AI | 88 | - | **8.36E-09** | 5.79E-02 |
| 2 | Fat | 88 | - | **4.25E-09** | 4.61E-02 |
| 3 | Pec | 44 | - | **7.40E-03** | **4.48E-04** |
| 3 | Gastro | 56 | - | 1.65E-02 | **7.39E-03** |
| 3 | Edl | 64 | - | **2.96E-03** | 1.57E-01 |
| 4 | IGF1 | 68 | - | **1.16E-03** | **1.48E-02** |
| 5 | Gastro | 49 | - | 6.30E-02 | 1.88E-02 |
| 6 | Gastro | 0 | - | 5.72E-02 | **1.97E-03** |
| 6 | AI | 27 | - | **1.21E-05** | 3.76E-01 |
| 6 | Fat | 28 | - | **1.40E-05** | 3.72E-01 |
| 6 | lengthNA | 45 | 1.83E-02 | **1.29E-04** | 1.76E-01 |
| 6 | lengthNT | 45 | - | **8.07E-07** | 2.19E-01 |
| 7 | Gastro | 47 | - | 1.53E-02 | 2.07E-02 |
| 7 | Pec | 47 | - | **1.34E-05** | 1.67E-01 |
| 8 | Gastro | 37 | - | **1.77E-04** | 6.04E-01 |
| 8 | Fat | 68 | - | **5.47E-08** | 8.94E-01 |
| 8 | AI | 69 | - | **5.28E-08** | 8.43E-01 |
| 9 | Gastro | 0 | - | 1.50E-01 | 1.11E-02 |
| 9 | Edl | 1 | **4.15E-06** | 9.18E-01 | 2.47E-01 |
| 9 | AI | 23 | - | **5.00E-04** | 1.66E-02 |
| 9 | Fat | 23 | - | **8.66E-04** | **7.91E-03** |
| 10 | lengthNT | 26 | - | **1.51E-04** | 2.33E-01 |
| 10 | Gastro | 30 | - | **3.93E-03** | 4.23E-01 |
| 10 | lengthNA | 58 | **7.99E-05** | 3.13E-02 | 1.41E-01 |
| 11 | lengthNA | 23 | 3.31E-02 | **2.91E-05** | 9.54E-02 |
| 11 | Tail | 25 | - | **9.29E-09** | 7.25E-01 |
| 11 | lengthNT | 26 | - | **1.53E-10** | 3.51E-01 |
| 11 | BMI | 49 | - | **1.41E-04** | 1.75E-01 |
| 11 | Gastro | 68 | - | **2.35E-03** | 5.13E-01 |
| 14 | Gastro | 34 | - | **1.12E-04** | 4.74E-01 |
| 17 | AI | 17 | - | **4.20E-06** | 5.85E-01 |
| 17 | Fat | 17 | - | **4.99E-06** | 4.98E-01 |
| 17 | Edl | 33 | - | **1.37E-04** | 3.64E-01 |
| 17 | Gastro | 68 | - | **1.50E-05** | 5.69E-01 |
| 18 | lengthNT | 34 | - | **3.85E-05** | 7.60E-01 |
| 18 | lengthNA | 35 | - | **5.06E-05** | 6.33E-01 |
| 18 | AI | 39 | 4.45E-02 | **1.36E-05** | 3.99E-01 |
| 18 | Fat | 39 | 4.00E-02 | **1.21E-05** | 4.02E-01 |

^a^ Trait abbreviations are the same as in Table 1.

^b^ Peak position of QTL detected in Kosambi centimorgans.

^c^ Comparison *P*-value for testing QTL effect. i: imprinting effect; a: additive effect; d: dominance effect. Only imprinted QTL (Table 3) were estimated for the imprinting effect. A “-“ notation indicates that the effect was not estimated. *P*-value< 0.01 is shown in bold type.

**Supplemental Table 5. List of primers used in the qPCR**

| Gene | Forward primer | Probe | Reverse primer | Product size |
| --- | --- | --- | --- | --- |
| Actb | GCTTCTTTGCAGCTCCTTCGT | CGGTCCACACCCGCCACCAG | ACCAGCGCAGCGATATCG | 76 |
| Adfp | GGTGATGGCAGGCGACAT | TACTCCGTATTCCGCAATGCTGCCTC | CGCCATCGGACACTTCCT | 68 |
| Atp2a2 | CCGCTACCTCATCTCATCCAA | AAGTGGTCTGTATCTTCCTGACGGCAGC | GACAGGAATTAAAGCCTCAGGAAA | 88 |
| EGF | GCTACGAAGGAGACGGGATCT | CTGTTTCGATATTGACGAGTGCCAGCG | TGCAGGCGGCATTCTCA | 84 |
| Igf1 | CCACACTGACATGCCCAAGA | TCAGAAGTCCCCGTCCCTATCGACAA | CCTTCTCCTTTGCAGCTTCGT | 76 |
| Igf2 | TCCCTTTGTCATCATGTGAAGACT | TGGCCCCAGGTGTTTGCCTCA | GAAAATGCATGTTAGAGGGACTGA | 126 |
| Myf5 | CCCTCCAGCTCCAGACTTATCTAT | TGCTAGG AGGGCGTCCTTCATGGA | GCTTGTCTTTCCTTCAGCTTCAG | 117 |
| Tnni1 | CCGGAAGTTGAGAGGAAATCC | CATGCTGAAGAGCCTGATGCTAGCCAAG | TTCCTGCTCCCAACACTCCTT | 96 |
| Wnt4 | AGCCGGGCACTCATGAATC | TCACAACAACGAGGCTGGCAGGAAG | CCCGCATGTGTGTCAAGATG | 67 |

Gene abbreviation: beta-actin (Actb), adipophilin (Adfp), ATPase2 (Atp2a2), epidermal growth factor (Egf), insulin-like growth factor 1 (IGF1), insulin-like growth factor 2 (Igf2), myogenic factor 5 (Myf5), troponinI (Tnni1) and wingless-related MMTV integration site 4 (Wnt4).

**Supplemental Table 6. List of 152 SNPs used in the final QTL mapping**

| SNP name | MMU | cM (Crimap)^a^ | cM^b^ | Map Location^c^ |
| --- | --- | --- | --- | --- |
| rs13475701^c^ | 1 | 0.101 | 0.101 | 4486478 |
| rs3696088 | 1 | 17.801 | 21.384 | 41127587 |
| rs13472794 | 1 | 28.501 | 36.051 | 67523394 |
| rs13475931 | 1 | 37.001 | 44.353 | 76665687 |
| rs3670389 | 1 | 41.801 | 51.993 | 88560363 |
| rs3667720 | 1 | 49.501 | 61.061 | 120679081 |
| rs3703729 | 1 | 54.101 | 71.657 | 134380222 |
| rs13476201 | 1 | 64.301 | 84.016 | 158969249 |
| rs13476229 | 1 | 71.301 | 93.679 | 169239352 |
| rs3658234 | 1 | 72.701 | 99.299 | 175004447 |
| rs3666905 | 1 | 81.701 | 112.130 | 187551710 |
| rs13476312 | 1 | 89.901 | 121.484 | 193706020 |
| rs13476334^c^ | 2 | 3.176 | 3.176 | 7291472 |
| gnf02.013.589 | 2 | 12.276 | 14.075 | 16612103 |
| gnf02.035.469 | 2 | 22.776 | 27.077 | 33926782 |
| rs6268714 | 2 | 30.676 | 35.464 | 57637194 |
| rs13476554 | 2 | 39.076 | 43.046 | 67097663 |
| rs6345656 | 2 | 46.476 | 48.890 | 74672761 |
| rs13476636 | 2 | 52.076 | 56.122 | 91349993 |
| rs3144393 | 2 | 68.676 | 70.425 | 118272621 |
| rs13476878 | 2 | 92.976 | 91.035 | 160226215 |
| rs3143843 | 2 | 101.676 | 98.041 | 169461615 |
| rs6335805 | 2 | 112.076 | 110.897 | 178995956 |
| rs13477019^c^ | 3 | 7.734 | 7.734 | 23242284 |
| rs3663409 | 3 | 12.434 | 15.107 | 31989790 |
| rs13477132 | 3 | 32.634 | 29.787 | 57754706 |
| rs13477174 | 3 | 38.734 | 34.080 | 68244840 |
| rs3670634 | 3 | 45.134 | 42.725 | 86937167 |
| rs3663873 | 3 | 54.834 | 56.769 | 109985432 |
| rs13477430 | 3 | 77.434 | 70.816 | 134895047 |
| rs3724562 | 3 | 99.434 | 84.649 | 150313284 |
| CEL-3_159340478 | 3 | 120.434 | 93.158 | 157721197 |
| rs13477622^c^ | 4 | 16.514 | 16.514 | 28506701 |
| rs6232550 | 4 | 26.414 | 26.916 | 41164426 |
| rs3725792 | 4 | 30.214 | 30.376 | 44148723 |
| rs3715031 | 4 | 36.214 | 37.502 | 54639377 |
| rs13477745 | 4 | 42.114 | 46.233 | 64477730 |
| rs3717837 | 4 | 50.114 | 54.794 | 85074942 |
| mCV23905937 | 4 | 57.014 | 64.559 | 98478276 |
| rs6324470 | 4 | 61.414 | 69.206 | 104937019 |
| rs3659226 | 4 | 87.114 | 93.423 | 140587789 |
| SNP name | MMU | cM (Crimap)^a^ | cM^b^ | Map Location^c^ |
| CEL-4_149694865 | 4 | 95.914 | 101.347 | 150301285 |
| CEL-5_5867251^c^ | 5 | 2.208 | 2.208 | 5866538 |
| UT_5_19.849706 | 5 | 11.308 | 9.503 | 20201136 |
| rs6256504 | 5 | 45.708 | 24.714 | 38515920 |
| CEL-5_52953963 | 5 | 55.108 | 33.321 | 53136372 |
| rs3707918 | 5 | 64.808 | 43.343 | 72127548 |
| rs3720626 | 5 | 67.908 | 50.292 | 76272435 |
| rs3706737 | 5 | 80.308 | 64.852 | 96528453 |
| rs13478466 | 5 | 86.608 | 73.634 | 111798781 |
| CEL-5_117736621 | 5 | 92.908 | 80.048 | 119400029 |
| gnf05.124.386 | 5 | 101.608 | 87.408 | 129543594 |
| rs3721911 | 5 | 114.408 | 96.544 | 138881660 |
| rs6284348 | 5 | 118.108 | 101.586 | 142681650 |
| rs13478595 | 5 | 132.008 | 110.300 | 149284218 |
| rs13478602^c^ | 6 | 0.001 | 0.001 | 3799841 |
| petM-02094-1 | 6 | 6.601 | 7.058 | 17707097 |
| rs3678887 | 6 | 12.701 | 13.010 | 32386962 |
| rs13478727 | 6 | 19.001 | 23.577 | 43991377 |
| rs13478839 | 6 | 32.001 | 39.724 | 78161749 |
| rs4226048 | 6 | 34.201 | 45.897 | 84113292 |
| mCV24115224 | 6 | 59.701 | 66.688 | 115076942 |
| UT_6_123.37228 | 6 | 64.401 | 72.189 | 122110793 |
| rs3688358 | 6 | 71.401 | 79.980 | 132429066 |
| rs3725987 | 6 | 76.501 | 84.123 | 138352837 |
| rs13479099 | 6 | 87.501 | 93.881 | 148127251 |
| mCV22975338^c^ | 7 | 11.749 | 11.749 | 22376177 |
| rs3719256 | 7 | 28.849 | 23.532 | 38614565 |
| rs3717846 | 7 | 36.749 | 30.339 | 58400141 |
| rs3676254 | 7 | 42.749 | 38.005 | 66569463 |
| rs3656205 | 7 | 47.649 | 45.440 | 75343354 |
| rs13479422 | 7 | 51.949 | 53.395 | 91892904 |
| rs13479471 | 7 | 57.449 | 65.046 | 108991781 |
| rs6275579 | 7 | 62.549 | 71.740 | 117862298 |
| rs13479657^c^ | 8 | 14.694 | 14.694 | 24618506 |
| rs13479757 | 8 | 46.894 | 29.437 | 50420731 |
| rs13479844 | 8 | 56.094 | 38.394 | 75473491 |
| rs3678433 | 8 | 60.394 | 45.510 | 83208899 |
| rs6182338 | 8 | 77.894 | 60.514 | 106277983 |
| rs13480071^c^ | 9 | 0.372 | 0.372 | 12385785 |
| rs13480109 | 9 | 6.172 | 8.209 | 25783299 |
| rs13480128 | 9 | 10.072 | 16.473 | 32666876 |
| rs3719607 | 9 | 13.872 | 23.433 | 37141308 |
| rs8259427 | 9 | 18.972 | 29.955 | 44998572 |
| SNP name | MMU | cM (Crimap)^a^ | cM^b^ | Map Location^c^ |
| rs6213724 | 9 | 38.772 | 52.130 | 79802975 |
| rs3712946 | 9 | 44.972 | 58.044 | 94386044 |
| rs3657881 | 9 | 51.972 | 68.561 | 105214342 |
| rs13480408 | 9 | 55.672 | 73.963 | 108844971 |
| rs13480429 | 9 | 65.272 | 81.464 | 113641808 |
| rs13480553^c^ | 10 | 10.818 | 10.818 | 25770673 |
| rs13480578 | 10 | 15.318 | 17.160 | 34640648 |
| CEL-10_58149652 | 10 | 30.018 | 27.700 | 58359416 |
| rs13480647 | 10 | 38.718 | 41.670 | 72833057 |
| rs3717445 | 10 | 42.218 | 46.578 | 82879911 |
| rs13480707 | 10 | 46.418 | 54.006 | 92301690 |
| rs13480754 | 10 | 55.818 | 62.804 | 107371198 |
| rs13480776 | 10 | 64.518 | 69.333 | 115607504 |
| rs13480797 | 10 | 70.418 | 76.898 | 121584553 |
| rs8259806 | 10 | 76.918 | 81.668 | 127093506 |
| rs13480837^c^ | 11 | 0.201 | 0.201 | 3781731 |
| rs6276300 | 11 | 11.701 | 15.322 | 27142847 |
| rs6199956 | 11 | 37.201 | 30.082 | 50498109 |
| rs13481054 | 11 | 41.701 | 36.195 | 59881071 |
| rs3701609 | 11 | 49.801 | 46.592 | 72989283 |
| rs8270290 | 11 | 64.701 | 64.638 | 97032985 |
| rs3653651 | 11 | 67.601 | 74.951 | 101967778 |
| rs13481216 | 11 | 71.901 | 82.153 | 107232603 |
| rs6407460 | 11 | 78.501 | 90.804 | 113210907 |
| CEL-11_118234030 | 11 | 84.801 | 96.663 | 118383994 |
| rs3657682^c^ | 12 | 7.183 | 7.183 | 15091125 |
| rs6225272 | 12 | 14.783 | 13.903 | 27459535 |
| rs3725854 | 12 | 38.283 | 43.151 | 77999170 |
| rs6288403 | 12 | 44.983 | 50.050 | 86374922 |
| rs6390948 | 12 | 61.583 | 66.828 | 104149763 |
| rs3713779 | 12 | 67.783 | 70.502 | 113105127 |
| rs13481780^c^ | 13 | 17.399 | 17.399 | 40759094 |
| rs3678784 | 13 | 46.999 | 32.379 | 61647402 |
| rs13482096^c^ | 14 | 7.498 | 7.498 | 21085575 |
| rs8251329 | 14 | 26.998 | 19.246 | 49481253 |
| rs3712401 | 14 | 35.698 | 26.516 | 61778903 |
| rs3709178 | 14 | 44.998 | 39.330 | 79100609 |
| rs13482404 | 14 | 66.798 | 60.779 | 116103438 |
| rs13482893^c^ | 17 | 11.306 | 11.306 | 13745716 |
| rs3719497 | 17 | 19.706 | 16.339 | 23308349 |
| rs3023442 | 17 | 25.306 | 22.288 | 31902550 |
| rs6395919 | 17 | 44.706 | 35.586 | 48827083 |
| rs6257479 | 17 | 64.606 | 50.679 | 68982152 |
| SNP name | MMU | cM (Crimap)^a^ | cM^b^ | Map Location^c^ |
| rs3663966 | 17 | 71.606 | 57.945 | 76523597 |
| rs13483140 | 17 | 78.506 | 64.412 | 82893669 |
| rs3696168 | 17 | 83.806 | 69.866 | 88281513 |
| rs13483233^c^ | 18 | 7.969 | 7.969 | 18327172 |
| rs3723947 | 18 | 14.569 | 16.515 | 30680245 |
| rs6313313 | 18 | 18.369 | 23.174 | 41881959 |
| rs3722312 | 18 | 24.869 | 30.130 | 52561869 |
| rs3670254 | 18 | 33.269 | 36.847 | 58103478 |
| rs3718618 | 18 | 39.369 | 51.926 | 69446841 |
| rs13483438 | 18 | 47.969 | 59.023 | 74453057 |
| rs6236348^c^ | 19 | 0.448 | 0.448 | 6000609 |
| gnf19.017.711 | 19 | 12.148 | 13.059 | 19251882 |
| rs13483569 | 19 | 18.748 | 20.231 | 23009796 |
| rs6237466 | 19 | 29.148 | 29.105 | 31688846 |
| rs3703185 | 19 | 34.548 | 36.175 | 38314921 |
| rs8257588 | 19 | 40.448 | 42.991 | 47414608 |
| mCV24736382 | 19 | 48.248 | 51.488 | 54414305 |
| rs3718998 | 19 | 56.848 | 57.857 | 60090097 |
| rs13483724^c^ | X | 14.914 | 14.914 | 30628639 |
| rs13483748 | X | 23.614 | 22.853 | 45251371 |
| CEL-X_91222960 | X | 39.414 | 43.283 | 94420742 |
| rs13483992 | X | 47.314 | 55.715 | 124104620 |
| rs13484003 | X | 49.214 | 60.816 | 127148857 |
| rs13484087 | X | 61.614 | 80.594 | 149574316 |

^a^ Position of markers in Kosambi centimorgans. The linkage map was built using Cri-map (Green and Crooks, 1990).

^b^ Position of markers in Kosambi centimorgans. This linkage map was from the Wellcome-CTC Mouse Strain SNP Genotype Set

(<http://www.well.ox.au.uk/mouse/INBREDS>).

^c^ Positions of the first marker on each chromosome are from the Wellcome-CTC Mouse Strain SNP Genotype Set.

Reference

Green P, Falls K, Crooks S: *Documentation for CRIMAP, Version 2.4.* St. Louis: Washington University School of Medicine; 1990.
